# Protease sensitivity and stress adaptation of bioactive peptide-producing lactic acid bacteria: functional implications for food biopreservation

**DOI:** 10.1186/s12896-025-01077-y

**Published:** 2025-11-25

**Authors:** Oluwabukola Atinuke Popoola, Abimbola Ayodeji Orukotan, Olubunmi Olaitan Agarry

**Affiliations:** 1 Genetics, Genomics and Bioinformatics Department, National Biotechnology Research and Development Agency (NBRDA), Abuja, Nigeria; 2 College of Basic Sciences, Lagos State University of Science and Technology, Ikorodu, Lagos, Nigeria; 3 Department of Microbiology, University of Abuja, Abuja, Nigeria; 4 Department of Microbiology, Kaduna State University, Kaduna, Nigeria

**Keywords:** Protease sensitivity, Stress adaptation, Lactic acid bacteria (LAB), Bioactive peptides, Food biopreservation

## Abstract

**Background:**

The search for safe, natural food preservatives has turned attention to antimicrobial peptide (AMP)-producing lactic acid bacteria (LAB). These AMPs, which are bioactive peptides of proteinaceous nature, inhibit a broad spectrum of foodborne pathogens. Their proteinaceous composition ensures safety and digestibility; however, their effectiveness depends on the physiological resilience of the producing LAB under food-relevant stresses and the susceptibility of the AMPs to proteolytic degradation.

**Results:**

The 13 AMP-producing LAB strains tolerated a wide pH range (4.5–8.5), multiple temperatures (20–45 °C), and moderate to high salt concentrations (5.5–10% NaCl), demonstrating robustness under diverse food processing and storage conditions. Even after exposure to these physiological stresses, the strains retained antimicrobial activity, producing zones of inhibition ranging from 5 mm under extreme stress conditions to 20 mm under optimum growth conditions against *Staphylococcus aureus* ATCC 25923 and *Escherichia coli* ATCC 25922. The antimicrobial peptides were completely inactivated by protease treatments with pepsin, trypsin, proteinase K, and papain, confirming their proteinaceous nature while highlighting protease susceptibility. Six of the 13 LAB strains had been previously 16S rRNA-sequenced (GenBank accession numbers PV983358–PV983363), including one strain showing 93.54% sequence identity to the closest known species; suggesting potential novelty.

**Conclusions:**

AMP-producing LAB from Nigerian non-dairy fermentations exhibit broad physiological adaptability and produce proteinaceous antimicrobial peptides with notable inhibitory activity against foodborne pathogens, even under stress conditions. Although complete protease susceptibility limits in vivo stability, their safety, traceable identification, and environmental robustness underscore their promise as natural, clean-label food preservatives, supporting the development of safe, minimally processed food strategies.

## Background

The growing consumer demand for safe, natural, and minimally processed foods has spurred considerable interest in microbial-derived bio-preservatives, which offer an alternative to synthetic chemical additives that may pose health concerns or alter sensory properties [1–3]. Among these natural preservatives, antimicrobial peptide (AMP)-producing lactic acid bacteria (LAB) have emerged as particularly promising due to their dual functionality: they inhibit a broad spectrum of foodborne pathogens and spoilage organisms, while remaining digestible and generally recognized as safe (GRAS) [4–7].

AMPs are small, proteinaceous compounds, often referred to as bacteriocin-like substances, that inhibit diverse microorganisms through multiple mechanisms, including disruption of cell membranes, interference with intracellular targets, and inhibition of essential enzymatic processes [8–11]. Their proteinaceous nature confers both safety and specificity, as they are typically degraded by digestive enzymes and do not accumulate in the body [4, 12]. This broad-spectrum antimicrobial activity, coupled with their environmental resilience, makes AMP-producing LAB ideal candidates for incorporation into minimally processed foods, where they can enhance safety and extend shelf life without compromising nutritional quality or organoleptic properties [13–15].

However, the effectiveness of AMPs in food systems is strongly influenced by the physiological resilience of the producing LAB, as environmental conditions such as pH fluctuations, temperature variations, and high salt concentrations can impact both microbial growth and AMP production [8, 16–19]. Furthermore, AMPs are often susceptible to proteolytic degradation, which may limit their functionality in protein-rich or enzyme-active foods [20–24]. While many studies have investigated AMP-producing LAB from dairy environments, where growth conditions are relatively stable, comparatively few have explored non-dairy fermented foods, which provide more dynamic and challenging ecological niches [25, 26].

Non-dairy fermented foods, common across Africa and Asia, often support diverse microbial communities that have evolved remarkable tolerance to fluctuating environmental and nutritional conditions, making them valuable sources of stress-resilient LAB strains [27–29].

In Nigeria, traditional non-dairy fermented foods such as soy milk, tigernut milk, and sorghum-based gruels are widely consumed as part of daily diets and are characterized by rich microbial diversity shaped by traditional processing methods [30, 31]. This makes them a promising reservoir for LAB strains capable of producing antimicrobial peptides with broad activity and stability under food-relevant conditions [14, 32, 33]. Exploring these traditional fermentations could therefore expand the repertoire of biopreservative candidates, especially those that combine safety, adaptability, and inhibitory activity against foodborne pathogens [34–38].

**Fig. 1:**
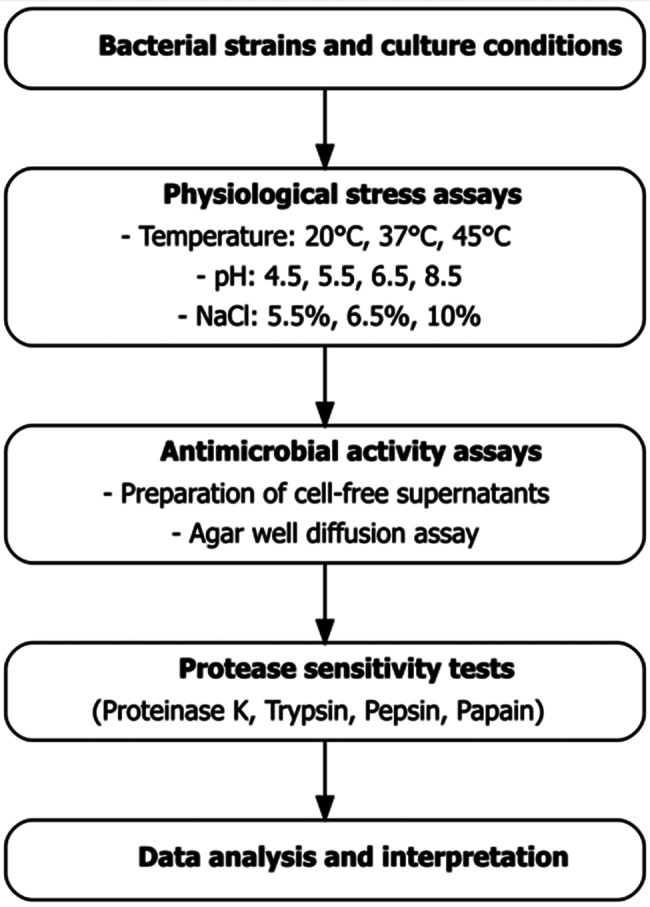
Experimental workflow of the study

This study evaluates the physiological adaptability and protease sensitivity of 13 AMP-producing LAB strains isolated from Nigerian non-dairy fermentations. These strains (SGA2, SGA12, SGB13, TNA7, TNA9, TNA10, TNA14, SGB11, SMA1, SMA12, SGA8, SMB5, and TNA4) were characterized based on their morphological, biochemical, and physiological properties, while six representative isolates (SGB11, SGA12, TNA9, SGA2, TNA14, and SMA1) were further identified through 16S rRNA gene sequencing (GenBank accession numbers PV983358–PV983363). By investigating their tolerance to food-relevant stresses and the stability of their AMPs, this work aims to assess their potential as natural, clean-label food preservatives, thereby expanding the understanding of LAB from traditional African fermentations as promising biotechnological resources. The workflow adopted in this study is illustrated in Fig. 1.

## Methods

### Bacterial strains and culture conditions

Thirteen AMP-producing LAB strains (SGA2, SGA12, SGB13, TNA7, TNA9, TNA10, TNA14, SGB11, SMA1, SMA12, SGA8, SMB5, and TNA4) were used in this study. These strains were originally isolated from Nigerian non-dairy fermented foods, including soy milk, tigernut milk, and sorghum gruel, using standard microbial isolation procedures [12]. Six of the strains were molecularly characterized through 16S rRNA sequencing (GenBank accession numbers PV983358–PV983363), including one putative novel strain currently undergoing whole-genome sequencing. All LAB strains were stored in 20% (v/v) glycerol at −20 °C. For experimental use, each isolate was revived by defrosting at 37 °C for 15 min followed by two successive transfers subcultures in MRS broth (ReadyMED Media, India) and incubated at 37 °C for 18–24 h under microaerophilic conditions (Memmert INE400, Germany). Active cultures were streaked onto MRS agar (ReadyMED Media, India) plates to verify purity, and single colonies were subsequently inoculated into fresh MRS broth [39].

### Physiological stress assays

The physiological adaptability of the LAB strains was evaluated under food-relevant stresses, including variations in pH, temperature, and salt concentration, with methods adapted from [40] with slight modifications. Briefly, actively growing cultures (5% v/v inoculum, 250 μL into 5 mL MRS broth) were incubated under each stress condition for 24 h.

For pH tolerance, MRS broth (ReadyMED Media, India) was adjusted to pH 4.5, 5.5, 6.5, and 8.5 using 1 N HCl or NaOH (analytical grade, Sigma-Aldrich, USA) before inoculation, and cultures were incubated at 37 °C. For temperature tolerance, inoculated MRS broth was incubated at 20, 37 and 45 °C. These temperatures were selected to represent food-relevant stress limits: 37 °C as the optimal growth temperature for most mesophilic LAB, 20 °C as a suboptimal (low-temperature) stress condition, and 45 °C as a supra-optimal (high-temperature) condition. Although 30 °C is often used for LAB cultivation, it falls within the near-optimal range and was therefore not used as a thermal stress threshold. For salt tolerance, MRS broth was supplemented with NaCl (analytical grade, Sigma-Aldrich, USA) at final concentrations of 5%, 6.5 and 10% (w/v) and incubated at 37 °C [40].

Bacterial growth under all conditions was monitored spectrophotometrically (L7 Double Beam UV-VIS Spectrophotometer, LabTech, UK) at OD₆₀₀ after 0, 3, and 6 h of incubation. These time points correspond to the early to mid-logarithmic growth phase of the LAB strains under the tested stress conditions, which provides the most informative assessment of their physiological adaptability.

### Antimicrobial activity assays

#### Preparation of cell-free supernatants from 13 AMP-producing LAB strains

To obtain cell-free supernatants (CFS), actively growing AMP-producing LAB cultures were prepared by inoculating 0.1 mL (1% v/v) of an overnight culture grown in 5 mL MRS broth into 10 mL of fresh MRS broth, followed by microaerophilic incubation at 37 °C for 18–24 h. Cultures were centrifuged at 6000 rpm for 15 min (Eppendorf Centrifuge 5702 Hamburg, Germany) to remove cells, and the resulting supernatants were passed through 0.22 µm syringe filters (mdi Membrane Technologies, USA). The pH was adjusted to 6.5–7.0 using 1 M NaOH. Filtrates were stored at 4 °C for short-term use and aliquoted into vials for storage at − 20 °C for medium-term use. Previous studies have demonstrated that storage at − 20 °C effectively preserves the activity of antimicrobial peptides without significant degradation [39].

### Confirmation of test organisms

The test organisms, *Staphylococcus aureus* ATCC 25923 and *Escherichia coli* ATCC 25922, were sub-cultured for purity and reconfirmed on their respective selective media: Mannitol Salt Agar for *S. aureus*, Eosin Methylene Blue (EMB) Agar for *E. coli* (all from Oxoid Ltd., Hampshire, UK).

### Agar well diffusion assay

The inhibitory activity of LAB strains against foodborne pathogens was assessed after exposure to physiological stress conditions using *Staphylococcus aureus* ATCC 25923 and *Escherichia coli* ATCC 25922 as test organisms. Pathogens were grown in Mueller–Hinton broth at 37 °C for 18 h, and the inocula were standardized to 0.5 McFarland (≈1.5 × 10^8^ CFU/mL). Standardized suspensions were spread on Nutrient agar plates, after which wells (6 mm diameter) were created with a sterile cork borer and filled with 100 μL of cell-free supernatant (CFS) from the LAB strains. Plates were held at room temperature for 2 h to allow diffusion and then incubated at 37 °C for 18–24 h. Antimicrobial activity was determined by measuring the diameter of the clear zones surrounding each well. Measurements were performed in triplicate, and the mean inhibition zone was reported. Zones ≥0.5 mm were considered positive for antimicrobial activity. Replicates refer to different wells inoculated with the same cell-free supernatant and test organism on separate plates to ensure consistency and reliability [41]. To evaluate the effect of stress on antimicrobial activity, inhibition zones obtained under extreme stress conditions (corresponding to minimal growth) were compared with those recorded under optimum conditions.

### Protease sensitivity tests

The proteinaceous character of the antimicrobial metabolites was examined by subjecting the cell-free supernatants (CFS) obtained from the 13 LAB strains to enzymatic degradation. The procedure was adapted from [42] with minor modifications. Specific enzyme–buffer systems were prepared as follows: trypsin (Sigma, Germany) in 40 mM Tris–HCl buffer (pH 8.2), pepsin (Merck, Germany) in 0.002 M HCl (pH 2.0), proteinase K (Sigma, Germany) in 10 mM Tris–HCl (pH 7.5), and papain (Sigma, Germany) in phosphate-buffered saline (PBS, pH 6.8). Equal volumes (1 mL) of CFS and the respective enzyme solution were combined, while buffer-only mixtures with CFS served as enzyme-free controls. Untreated AMPs were included as positive controls. After incubation at 37 °C for 2 h, the enzyme activity was terminated by heating the mixtures in a boiling water bath for 5 min. The treated supernatants were subsequently tested for antimicrobial activity against *Staphylococcus aureus* ATCC 25923 and *Escherichia coli* ATCC 25922 using the agar well diffusion method as outlined earlier. The disappearance of inhibitory zones following enzyme exposure was taken as confirmation that the active compounds were proteinaceous in nature.

### Statistical analysis

All assays were conducted in triplicate, and results are expressed as mean ± standard deviation. Variations in inhibition zones among strains and treatment conditions were assessed using one-way analysis of variance (ANOVA) with Tukey’s HSD post hoc test at a significance level of *p* < 0.05. Statistical analyses were performed in R version 4.5.1 using the agricolae package, while graphical representation of data, including bar plots with error bars and significance annotations, was generated with ggplot2 and ggpubr packages [43].

## Results

### Antimicrobial peptide-producing bacterial strains

Thirteen AMP-producing LAB strains (SGA2, SGA12, SGB13, TNA7, TNA9, TNA10, TNA14, SGB11, SMA1, SMA12, SGA8, SMB5, and TNA4) were used in this study. Table 1 provides detailed information on each strain, including source of isolation, identification method, isolate name, and NCBI accession numbers (for the six 16S rRNA-characterized strains). The strains were subjected to physiological stress conditions (temperature, pH, and NaCl) and protease sensitivity assays to evaluate the stability and proteinaceous nature of their antimicrobial peptides. Their antimicrobial activity against *Staphylococcus aureus* ATCC 25923 and *Escherichia coli* ATCC 25922 was further confirmed following exposure to stress conditions.

**Table 1:** Previously characterized AMP-producing lactic acid bacteria used in this study

Strain code	Source of isolation	Identifiaction method	Isolate Name	NCBI accession number
SGA2	Fermented sorghum gruel	Phenotypic+16S rRNA	Limosilactobacillus fermentum	PV983361
SGA12	Fermented sorghum gruel	Phenotypic+16S rRNA	Lactiplantibacillus plantarum	PV983359
SGB13	Fermented sorghum gruel	Phenotypic	Lactiplantibacillus sp. (Presumptive)	-
TNA7	Fermented tigernut milk	Phenotypic	Lactiplantibacillus sp. (Presumptive)	-
TNA9	Fermented tigernut milk	Phenotypic+16S rRNA	Limosilactobacillus fermentum	PV983360
TNA10	Fermented tigernut milk	Phenotypic	Limosilactobacillus sp. (Presumptive)	-
TNA14	Fermented tigernut milk	Phenotypic+16S rRNA	Limosilactobacillus fermentum	PV983362
SGB11	Fermented sorghum gruel	Phenotypic+16S rRNA	Lactiplantibacillus plantarum	PV983358
SMA1	Fermented soy milk	Phenotypic+16S rRNA	Limosilactobacillus fermentum	PV983363
SMA12	Fermented soy milk	Phenotypic	Limosilactobacillus sp. (Presumptive)	-
SGA8	Fermented sorghum gruel	Phenotypic	Limosilactobacillus sp. (Presumptive)	-
SMB5	Fermented soy milk	Phenotypic	Lactiplantibacillus sp. (Presumptive)	-
TNA4	Fermented tigernut milk	Phenotypic	Limosilactobacillus sp. (Presumptive)	-

### Effect of physiological stress on AMP-producing LAB

#### Growth of AMP-producing LAB strains at different temperatures

**Fig. 2:**
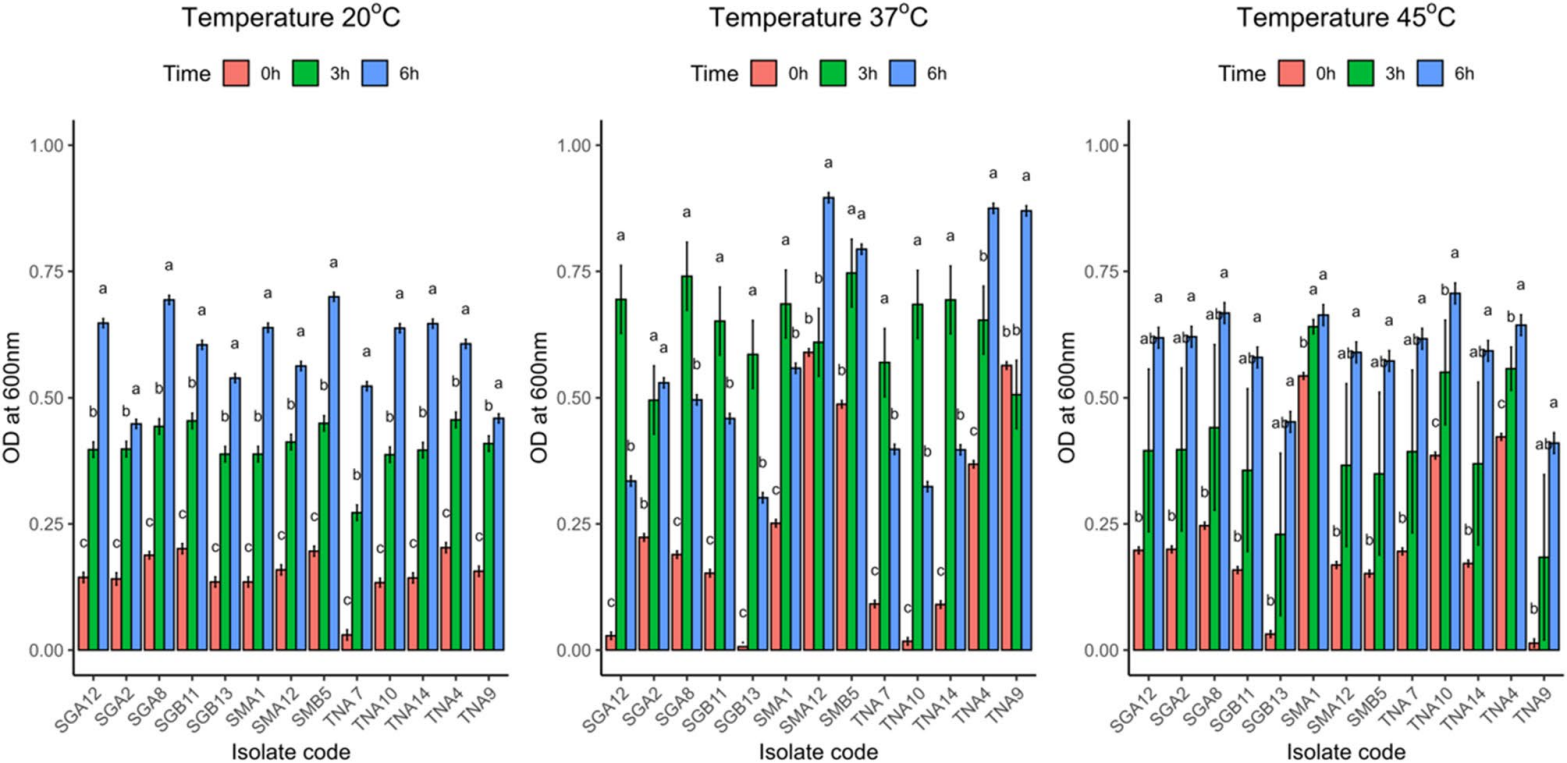
(**a–c**) growth of bioactive peptide-producing lactic acid bacteria at different temperatures (**a**: 20 °C, **b**: 37 °C, and **c**: 45 °c). Error bars represent standard deviation (*n* = 3). Bars sharing the same letter are not significantly different (*p* > 0.05) according to Tukey’s honest significant difference (HSD) test following one-way ANOVA

The thirteen AMP-producing LAB strains exhibited differential growth at 20 °C, 37 °C, and 45 °C°C, indicating variable tolerance to thermal stress (Fig. 2a-c).

The growth responses of AMP-producing LAB strains varied across temperatures (20 °C, 37 °C, and 45°C°C). At 20 °C, most isolates exhibited slow initial growth with gradual increases, reflecting limited metabolic activity, although SGA8 and SMB5 showed relatively higher OD values, suggesting better cold adaptation than strains such as TNA7. At 37 °C, growth was generally maximal, confirming this as the optimum temperature, with SMA12, TNA4, and SMB5 achieving the highest OD values, whereas SGA12 and TNA10 declined slightly after 6 h, possibly due to transient stress. At 45 °C, growth was more variable; TNA10 and SGA2 maintained appreciable OD values, indicating elevated temperature tolerance, while TNA9 showed marked reduction. These findings demonstrate strain-dependent thermal adaptability, highlighting certain isolates with broader tolerance ranges that may be advantageous for food preservation applications.

#### Growth of AMP-producing LAB under different NaCl concentrations

**Fig. 3:**
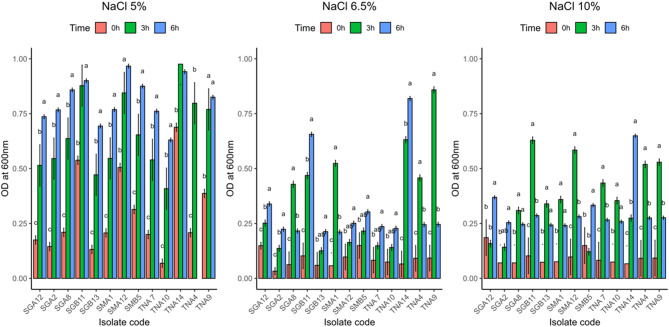
(**a–c**) Growth of antimicrobial peptide-producing bacteria at different salt concentrations (**a**: 5%, **b**: 6.5%, **c**: 10%). Error bars represent standard deviation (*n* = 3). Bars sharing the same letter are not significantly different at *p* > 0.05, according to Tukey’s Honest Significant Difference (HSD) test following one-way ANOVA

The thirteen AMP-producing LAB strains exhibited variable tolerance to increasing NaCl concentrations (Fig. 3a–c). Growth was optimal at 5% NaCl, with all isolates showing active multiplication and several, particularly TNA4, SMA12, and TNA14, reaching optical densities above 0.9 after 6 h. Moderate inhibition occurred at 6.5% NaCl, where only a few strains such as TNA7 and TNA14 maintained appreciable growth, while others (e.g., TNA9 and TNA10) declined slightly after 3 h. At 10% NaCl, growth was markedly suppressed across all isolates, with only SGB11 and TNA14 retaining moderate tolerance (OD ≈ 0.6). These results indicate that most AMP-producing LAB strains are halotolerant up to 5–6.5% NaCl, but exhibit significant growth inhibition beyond this threshold.

#### Growth of AMP-producing LAB strains at different pH

**Fig. 4:**
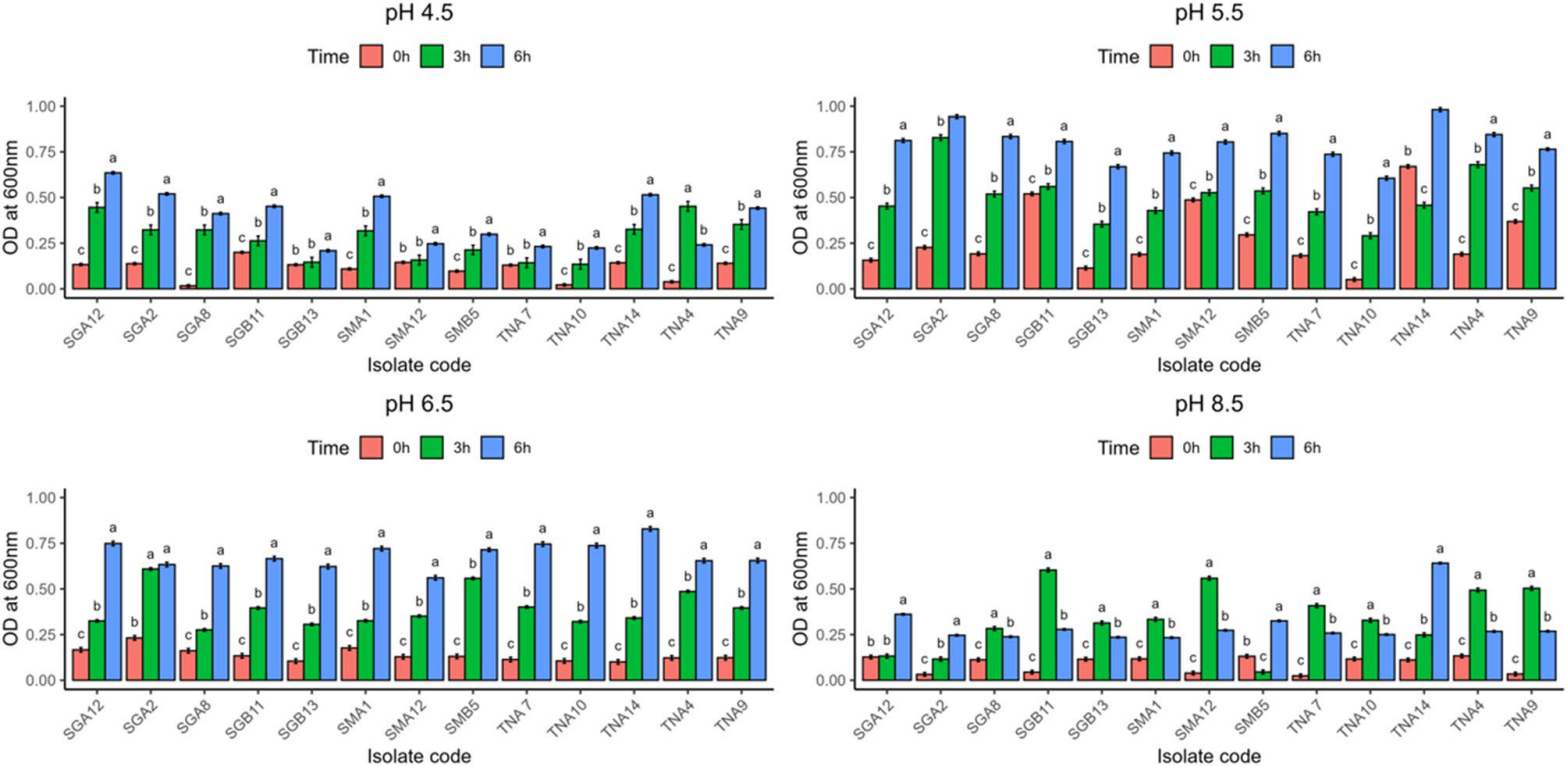
(**a–c**) Growth of antimicrobial peptide-producing bacteria at different pH (**a**: 4.5, **b**: 5.5, **c**: 6.5, **d**: 8.5). Error bars represent standard deviation (*n* = 3). Bars sharing the same letter are not significantly different at *p* > 0.05, according to Tukey’s Honest Significant Difference (HSD) test following one-way ANOVA

The AMP-producing LAB strains exhibited distinct growth patterns under varying pH conditions (Fig. 4a–d). Growth was most favourable at mildly acidic to neutral pH levels (5.5–6.5), where all strains showed active multiplication, particularly TNA14 and SGA2, which reached the highest OD values after 6 h. At pH 4.5, growth was notably reduced, though SGA12 and SGB13 maintained moderate tolerance, suggesting acid adaptation potential. Under alkaline conditions (pH 8.5), growth was significantly inhibited across most isolates, with only TNA14 and SGA12 displaying appreciable viability. These results indicate that most strains prefer near-neutral environments, with limited adaptability to strong acidic or alkaline stress.

#### Antimicrobial activity of 13 AMP-producing LAB strains under physiological stress on S. aureus and E. coli

**Fig. 5:**
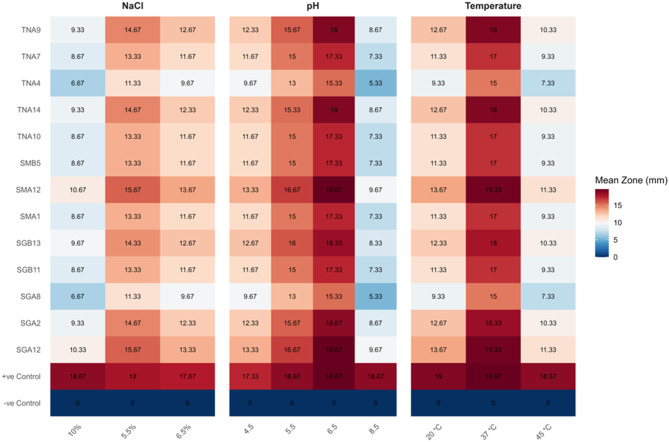
Heat map for antimicrobial activity of 13 AMP-producing LAB strains under physiological stress on *S. aureus*. Values represent the mean of three independent replicates (*n* = 3). Statistical analysis was performed using one-way ANOVA followed by Tukey’s HSD, but significance letters are not shown on the heat map

**Fig. 6:**
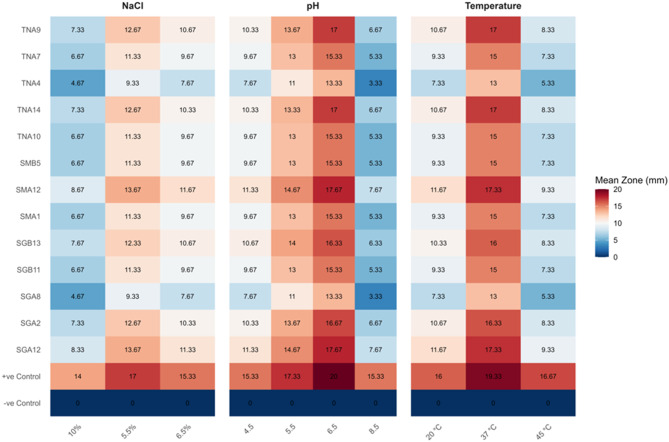
Heat map for antimicrobial activity of 13 AMP-producing LAB strains under physiological stress on *E. coli*. Values represent the mean of three independent replicates (*n* = 3). Statistical analysis was performed using one-way ANOVA followed by Tukey’s HSD, but significance letters are not shown on the heat map

To evaluate the antimicrobial activity of cell-free supernatants (CFS) obtained from lactic acid bacteria (LAB) under physiological stress, we assessed their activity against *Staphylococcus aureus* ATCC 25923 and *Escherichia coli* ATCC 25922. The LAB strains were subjected to stress conditions including varying temperature, pH, and NaCl concentrations. Heat map analysis revealed strain-specific differences, highlighting the variable capacity of individual LAB strains to maintain inhibitory activity under these stress conditions (Figs. 5 & 6).

#### Heat map analysis of antimicrobial activity of 13 LAB strains under temperature, pH and NaCl stress on S. aureus and E. coli

Heat map analysis revealed strain-specific variations in LAB-derived AMP activity under thermal stress. At 37 °C, most AMPs maintained strong inhibitory activity against *S. aureus* (13–19 mm) and *E. coli* (13–17 mm). Exposure to elevated temperature (45 °C) led to a notable reduction in activity, particularly for strains TNA4 and SGA8, while SGA12, SGA2, TNA9, TNA14, SMA12, and SGB13 retained higher inhibitory effects. At 20 °C, activity was moderately reduced, indicating some cold sensitivity.

AMP stability across pH 4.5–8.5 was generally maintained, with the strongest activity observed around neutral pH 6.5. Activity declined at pH 8.5, especially for *S. aureus*, while acidic conditions (pH 4.5–5.5) moderately reduced inhibition zones. Untreated AMPs (positive controls) maintained full activity, confirming intrinsic peptide stability.

Similarly, NaCl stress affected AMP activity in a concentration-dependent manner. At 5.5% NaCl, most AMPs showed strong inhibition against both pathogens. Moderate reductions were observed at 6.5% NaCl, and the highest salt stress (10% NaCl) caused a further decline, though some strains retained appreciable activity. These observations highlight the differential resilience of LAB-derived AMPs under thermal, pH, and osmotic stresses.

#### Protease sensitivity of AMPs

The antimicrobial activity of all the thirteen LAB-derived AMPs was completely abolished following treatment with proteinase K, trypsin, pepsin and papain. This indicates that the active compounds are entirely proteinaceous in nature. Untreated controls retained full activity, confirming that the observed inhibition was due to the AMPs (Table 2).

**Table 2:** Effect of protease treatment on the antimicrobial activity of 13 LAB-derived AMPs against *S. aureus* ATCC 25923 and *E. coli* ATCC 25922

LBA Strain	Proteinase K	Trypsin	Papain	Papain	Untreateed AMP on S. aureus	Untreated AMO on E.coli	% Activity Retained
SGA12	0	0	0	0	14± 1^bc^	15± 1^a^	0%
SGA2	0	0	0	0	14± 1^bc^	13± 1^abc^	0%
SGA8	0	0	0	0	13± 1^bcd^	11± 1^de^	0%
SGA11	0	0	0	0	15± 1^ab^	11± 1^de^	0%
SGA13	0	0	0	0	13± 1^bcd^	13± 1^abc^	0%
SMA1	0	0	0	0	11± 1^de^	12± 1^cde^	0%
SMA12	0	0	0	0	14± 1^bc^	11± 1^de^	0%
SMB5	0	0	0	0	16± 1^a^	11± 1^de^	0%
TNA 10	0	0	0	0	9± 1^e^	13± 1^bcd^	0%
TNA 7	0	0	0	0	12± 1^cd^	10± 1^e^	0%
TNA 9	0	0	0	0	13± 1^bcd^	14± 1^ab^	0%
TNA14	0	0	0	0	14± 1^bc^	12± 1^bcde^	0%
TNA4	0	0	0	0	12± 1^cd^	10± 1^e^	0%

Enzyme-treated AMPs (Proteinase K, Trypsin, Pepsin, Papain) showed no antimicrobial activity (0 mm zone of inhibition), indicating complete loss of activity. Untreated AMP served as the positive control (full activity) and was measured in triplicate. % Activity Retained represents enzyme-treated activity relative to the mean of the untreated control. All 13 LAB-derived AMPs were tested against S. aureus ATCC 25923 and E. coli ATCC 25922, with consistent results across both organisms. Superscript Tukey letters indicate statistically significant differences among untreated AMP values (p < 0.05)

Enzyme-treated AMPs (Proteinase K, Trypsin, Pepsin, Papain) showed no antimicrobial activity (0 mm zone of inhibition), indicating complete loss of activity. Untreated AMP served as the positive control (full activity) and was measured in triplicate. % Activity Retained represents enzyme-treated activity relative to the mean of the untreated control. All 13 LAB-derived AMPs were tested against *S. aureus* ATCC 25923 and *E. coli* ATCC 25922, with consistent results across both organisms. Superscript Tukey letters indicate statistically significant differences among untreated AMP values (*p* < 0.05).

## Discussion

This study evaluated the stability and functionality of antimicrobial peptides (AMPs) produced by thirteen LAB strains under different physiological stress conditions and protease treatments. The observed antimicrobial activity against *Staphylococcus aureus* and *Escherichia coli* even after exposure to temperature, pH, and NaCl stress highlights the resilience of these peptides and their potential application as natural biopreservatives in food systems, consistent with previous reports demonstrating the robustness of LAB-derived AMPs under environmental fluctuations [44–48]. The sensitivity of the antimicrobial activity to proteolytic enzymes further confirmed the proteinaceous nature of the bioactive compounds, in agreement with studies showing that bacteriocins and related LAB peptides are inherently proteinaceous [49–52]. Collectively, these observations support referring to the AMPs detected in this study as bacteriocin-like peptides, while noting that purification and structural characterization were not performed.

The ability of AMPs to retain activity under moderate stress conditions is particularly relevant for food processing and storage, where bioactive compounds face fluctuating environments. Strain-specific variability suggests that the structure of individual peptides contributes differently to stress tolerance, offering opportunities for targeted applications in food preservation and safety enhancement [53–55, 55–57].

Temperature significantly influenced LAB growth and metabolite production. Most strains grew optimally at 37 °C, with SMA12, TNA4, and SMB5 showing the highest optical densities, consistent with mesophilic LAB behavior [58–61]. Notably, TNA10, SGA2, and TNA7 maintained growth at 45 °C, reflecting thermotolerance comparable to *Lactobacillus plantarum* and *Enterococcus faecium*, advantageous for industrial applications [62–65]. Reduced growth at 20 °C in some strains indicates limited psychrotolerance, though SGA8 and SMB5 exhibited potential for chilled food applications [66–69]. The strain-specific variability observed likely reflects differences in stress-response systems, including heat-shock proteins, membrane adaptation, and regulation of bacteriocin gene clusters [70, 71]. These observations indicate that isolates with dual mesophilic and thermophilic adaptability are promising candidates for targeted food biopreservation.

AMP-producing LAB exhibited differential growth under varying NaCl concentrations, reflecting strain-specific salt tolerance. Robust growth at moderate salinity (5% NaCl) decreased at higher concentrations (6.5–10% NaCl), consistent with previous studies on LAB adaptation to saline environment. For instance, bacterial isolates from saline environments are promising candidates for fermentation of saline substrates, allowing high product yields without the need for prior desalination [72]. Similarly, certain LAB strains, such as *Lactobacillus acidophilus* CM1 and *Lactobacillus delbrueckii* OS1, can survive at 4–6% NaCl by synthesizing compatible solutes and modifying their membrane properties, reflecting their ability to adapt to high salinity [73]. The observed variability in growth responses among the LAB strains suggests that salt tolerance is strain-specific, influenced by genetic and physiological factors, which has implications for strain selection in food fermentation under high-salinity conditions [58, 74–76]. Further studies are warranted to elucidate the molecular mechanisms underlying salt tolerance in LAB and to explore the potential of these strains in producing bacteriocin-like peptides under saline conditions.

Growth across pH 4.5–8.5 revealed optimal activity at pH 5.5–6.5, with strain-specific differences highlighting the importance of selecting robust LAB for fermentation and AMP production under variable pH conditions [77]. These variations highlight the importance of selecting robust LAB for fermentation and AMP production under variable pH conditions, particularly for applications in foods with fluctuating acidity. Understanding these differences is essential for maximizing the stability and antimicrobial efficacy of bacteriocin-like peptides in diverse food matrices [78, 79].

Heat map analysis demonstrated that most AMPs maintained strong inhibitory activity at 37 °C, with moderate reduction at 45 °C and 20 °C°C. Activity was maximal at pH 6.5 and remained effective under NaCl concentrations of 5–10%, though inhibition declined at higher salinity. These findings underscore the potential of specific LAB strains to produce bacteriocin-like peptides with stability across practical stress ranges, supporting their use in food preservation and industrial applications [25, 80–85]. While the tested ranges (20–45 °C, pH 4.5–8.5, NaCl 5–10%) demonstrated AMP stability under practical conditions, broader stress gradients could further define extreme tolerance limits, which represents a limitation and a future direction for extending the applicability of these LAB strains in diverse food systems.

Complete inactivation of all AMPs by proteinase treatments confirms their proteinaceous nature, differentiating them from non-protein antimicrobials such as organic acids. This protease sensitivity also presents opportunities to enhance bacteriocin-like peptides applicability through protective delivery strategies, including encapsulation, nano-formulation, or immobilization [86–89]. The identification of strains that retain AMP activity under multiple stresses highlights candidates for further development as natural, strain-specific biopreservatives in the food industry.

## Conclusion

This study demonstrates that antimicrobial peptide (AMP)-producing lactic acid bacteria (LAB) isolated from Nigerian non-dairy fermented foods exhibit distinct physiological adaptations to temperature, pH, and osmotic stress, with strain-specific variations in growth and peptide activity. Several strains maintained robust antimicrobial activity under thermal, acidic, alkaline, and high-salinity conditions, with activities confirmed to be due to the proteinaceous peptides by neutralization of the supernatant, rather than acidity or alkalinity. The complete loss of activity upon protease treatment further supports their classification as bacteriocin-like compounds. These findings suggest that these LAB strains are promising natural antimicrobial agents for enhancing food safety and shelf-life in diverse processing environments. However, as only cell-free supernatants were tested and the peptides were not purified or structurally characterized, these conclusions are preliminary. Further analyses, including purification, structural characterization, and testing under broader stress conditions, are necessary to validate these observations and confirm their applicability across different food systems.

## Data Availability

The 16S rRNA gene sequences of the six AMP-producing LAB isolates used in this study are deposited in the NCBI GenBank repository under accession numbers PV983358–PV983363. Additional datasets generated and analyzed during the current study are available from the corresponding author upon reasonable request.
